# An Artificial Intelligence QRS Detection Algorithm for Wearable Electrocardiogram Devices

**DOI:** 10.3390/mi16060631

**Published:** 2025-05-27

**Authors:** Zihao Li, Wenliang Zhu, Yiheng Xu, Yunbo Guo, Junbo Li, Peng Song, Ying Liang, Binquan You, Lirong Wang

**Affiliations:** 1 School of Electronics and Information Technology, Soochow University, Suzhou 215006, China; 20234228017@stu.suda.edu.cn (Z.L.); 20234228023@stu.suda.edu.cn (Y.L.); 2 Suzhou Institute of Biomedical Engineering and Technology, China Academy of Sciences, Suzhou 215163, China; guoyb@sibet.ac.cn (Y.G.); lijb@sibet.ac.cn (J.L.); songp@sibet.ac.cn (P.S.); 3 School of Computer and Software Engineering, Huaiyin Institute of Technology, Huaian 223003, China; yiheng0106@163.com; 4 Suzhou Kowloon Hospital, Shanghai Jiao Tong University School of Medicine, Suzhou 215012, China; youbinquan@126.com

**Keywords:** multiple leads, QRS detector, scaling operation, universal

## Abstract

At the core of AI-driven electrocardiogram diagnosis lies the precise localization of the QRS complex. While QRS detection methods for multiple leads have been researched adequately in the last few decades, their multi-lead strategies still need to be designed manually. Therefore, a QRS detector that can fuse multiple leads automatically is still worth investigating. Methods: The proposed QRS detector comprises a leads-distillation module (LDM) and a QRS detection module. The LDM can distill multi-lead signals into single-lead ones. This procedure minimizes the weight proportions assigned to noisy leads, enabling the network to generate a novel signal that facilitates the recognition of QRS waves. The QRS detection module, utilizing U-Net, is capable of discerning QRS complexes from the novel signal. Results: Our method demonstrates outstanding performance with a parameter count of only 5216. It achieves an excellent F1 score of 99.83 on the MITBIHA database and 99.77 on the INCART database, specifically in the inter-patient pattern. In the cross-database pattern, our approach maintains a strong performance with an F1 score of 99.22 on the INCART database and an F1 score of 99.09 on the MITBIHA database. Conclusion: Our method provides a novel idea for universal multi-lead QRS detection. It possesses advantages, such as reduced computational parameters, enhanced precision, and heightened compatibility. Significance: Our method canceled the repeated deployment of the QRS detection function to different lead configurations in the electrocardiogram (ECG) diagnostic system. Moreover, the scaling operation may become a simple tool to decrease the computational load of the network.

## 1. Introduction

In recent years, despite the rapid growth and widespread integration of wearable devices into various facets of mainstream life and healthcare, the industry is still in the early stages of development, with many technologies still in research and development and not yet fully applicable in medical settings. Presently, wearable medical devices primarily concentrate on data collection, with limited capabilities in terms of data analysis, often requiring remote diagnosis from medical professionals. This presents challenges in terms of healthcare resources, with the scarcity of medical professionals impeding the realization of the full medical potential of wearable devices [1]. The advancement of artificial intelligence provides a promising solution to this challenge. In the future, AI-based diagnosis is poised to become a crucial foundation of healthcare [2].

In the post-pandemic era, wearable electrocardiogram (ECG) devices are experiencing a golden period of development. Indeed, it is a great opportunity to apply AI diagnosis in the field of wearable ECG devices. For wearable electrocardiogram (ECG) devices, their primary function is to upload the collected ECG data to the server via wireless or wired means. The server then processes the received data. The first challenge encountered in this processing is the accurate localization of the QRS complex. The QRS complex represents the electrical activity associated with ventricular depolarization, and its precise localization is crucial for ECG analysis. For instance, calculating classical electrocardiogram parameters, such as heart rate measurement, heart rate variability calculation, and RR interval measurement [3], is inherently intertwined with the precise localization of the QRS complex. Furthermore, when an AI-based application, such as a practical arrhythmia classifier, is centered around QRS complex detection, it often demonstrates superior interpretability, robustness, and accuracy compared to alternative methods [4,5]. Hence, the positional information of the QRS complex serves as the foundation for electrocardiogram diagnosis, and accurate localization of the QRS complex holds immense significance.

By localizing the QRS complex, more representative input features, such as QRS waveform and temporal characteristics, can be provided to deep learning models, and the effects of baseline drift, muscle interference, and other factors on the algorithm can be suppressed. Furthermore, it assists in preprocessing the ECG data, such as segmentation, normalization, alignment, etc., thereby improving the efficiency and accuracy of the algorithm. For example, Jinghao Niu et al. proposed a single-heartbeat classifier with an overall accuracy of 96.4% [4]. It takes symbolized heartbeat signals and RR intervals as the input and can separate heartbeats into the following five classes: non-ectopic, supraventricular ectopic, ventricular ectopic, fusion, and unknown. The overall accuracy of their classifier falls from 96.4% to 92.4% when missing the RR intervals. Our previous work proposed a heartbeat identifier that can identify ectopic heartbeats from long signals [5]. Our identifier needs to convert RR intervals to an auxiliary waveform, then take it and a long signal as the input. The experimental result shows that the F1 score for identifying ectopic heartbeats falls from 87.18 to 73.95 without the auxiliary waveform.

Over the past few decades, QRS detection methods have undergone extensive research. Notably, most of these methodologies developed by researchers are tailored for single-lead ECG signals. This focus has resulted in a prevalent scenario where the majority of wearable ECG diagnostic systems exhibit performance limitations or instability. Specifically, unforeseen noise interference or electrode detachment can significantly degrade the processing system’s effectiveness [6]. This is not conducive to the further development of wearable ECG devices. Furthermore, in clinical practice, ECG diagnostic systems typically employ between two and twelve leads. Consequently, leveraging the complementary information from multiple leads to enhance QRS complex detection has emerged as a critical safeguard for advancing the reliability of wearable ECG diagnostic systems. Currently, there are two primary approaches for multi-lead QRS complex detection, as follows:

(1) Selecting a signal with the best signal quality from several leads using various strategies, then using the single-lead QRS detection algorithm.
(2) Using the single-lead QRS detection algorithm on each lead, then fusing the detecting result of each lead using various strategies.

The first of the two methods above entails detecting QRS complexes after selecting a single lead. This selection process necessitates careful consideration of the noise distribution. Additionally, any change in the number of input leads alters the characteristics and available information of the input signals, often requiring manual adjustment of the lead selection strategy at the front end. As for the second method, variations in the number of input leads render the original fusion logic ineffective, necessitating manual modification of the fusion strategy. Both approaches, which rely on manual strategy adjustments to accommodate multi-lead fusion and QRS complex detection, inherently increase complexity and compromise generalizability.

In this paper, we propose a novel QRS detector based on the convolutional neural network for multi-lead ECG signals. Our approach can automatically fuse useful information from multiple leads without manual strategies and can be applied to multi-lead configurations [7] without any modification. The multi-lead configurations include the following:

(1) Six-lead configuration: I, II, III, avR, avL, and avF;
(2) Four-lead configuration: I, II, III, and V2;
(3) Three-lead configuration: I, II, and V2;
(4) Two-lead configuration: I and II;
(5) Single-lead configuration: II.

The proposed QRS detector mainly consists of two components, namely a leads-distillation module (LDM) and a QRS detection module.

The LDM is designed to adapt to multiple lead configurations, efficiently extracting useful information from each lead and integrating it into a unified single-lead signal. The QRS detection module is specifically engineered to identify QRS complexes from this integrated signal. To further enhance QRS complex detection performance, we implemented a downsampling operation and an upsampling operation to handle the input signal and network output, respectively. Collectively referred to as the “scaling operation,” these steps optimize feature extraction and signal reconstruction for more accurate QRS detection.

The main contributions of this study are the following aspects:

(1) We propose a novel multi-lead QRS detector based on convolutional networks. When the input leads change, the detector can be applied to multi-lead configurations without any strategy modification. In the relevant experiments, the performance of this method is superior to or comparable with that of the compared model.
(2) We discovered and explained a phenomenon, i.e., why the scaling operation can enhance the network’s ability to detect QRS complexes. In a variety of different experiments, this operation has led to a consistent improvement in sensitivity (se), positive predictive value (PPv), and F1 score.

The remainder of this work is organized as follows. Section 2 summarizes and lists related works. Section 3 describes the details of the proposed QRS detection approach. Section 4 describes the experimental results on different databases and different lead configurations. In Section 5, we discuss and analyze the phenomenon we found. Finally, we conclude this work and describe our future work.

## 2. Related Works

Compared with single-lead ECG signals, multi-lead ECG signals can provide more effective information in the presence of noise interference. As a result, an increasing number of researchers have dedicated themselves to developing multi-lead QRS complex detection algorithms, giving rise to numerous excellent detection algorithms.

Mariano Llamedo et al. proposed a method used to select the most suitable lead for performing heartbeat detection in multi-lead ECG signals and obtained a good result [8]. However, due to the heterogeneous distribution of noise, especially the electrode moving interference and muscle artifact [9], such a method may discard a bad signal which contains clear portions or choose a good signal which has noisy portions.

Maxime Yochum et al. proposed the automatic detection of P, QRS, and T patterns in 12-lead ECG signals [10]. Firstly, their method used the continuous wavelet transform (CWT) to calculate the CWT coefficients of a single-lead ECG signal with the mother wavelet “Daubechie 3” and the scale factor “38”. Secondly, they generated a mask to separate QRS waves from the original single-lead ECG signal according to the automatic threshold calculated from the histogram of the CWT coefficients. Finally, they summed all masks of 12 signals and regarded the average of summed masks as a decision threshold. If the value of a portion of the summed mask exceeds the decision threshold, then the portion corresponds to a QRS complex. Their approach can improve the performance of detecting QRS complexes by using other leads in particular when a lead has failed. Furthermore, their approach has a positive predictive value of 91.75% and an accuracy of 98.64% on the CinC Challenge 2011 database. Additionally, by extracting information from multi-lead signals, the noise can also be restrained. Therefore, it is significant to extract information from multi-lead signals.

Moreover, a couple of other works improve the QRS detection performance by fusing the results of the single-lead QRS detector. Carlos A. Ledezma et al. proposed a data fusion method to improve the performance of QRS detectors on multi-lead ECG signals [6]. Their method applied a single-lead algorithm on each channel of 12-lead signals and trained weights and thresholds to judge if an effective QRS complex was detected by the algorithm within a 150 ms window. They evaluated their method on the MIT-BIH arrhythmia database and the INCART database in conjunction with six single-lead algorithms. The result shows that the multi-lead detector achieves better performance than the corresponding single-lead detector on any given channel.

Chuang Han et al. proposed a method based on the U-Net framework to locate QRS complexes and T waves [11]. Also, they proposed a strategy to combine results coming from two heterogeneous U-Net frameworks to obtain the final detection result. Their method has high sensitivity and positive predictive value ranging from 99.45% to 100% for locating QRS complexes from three databases.

Existing research has conclusively demonstrated that multi-lead ECG signals offer enhanced useful information and resilience against noise interference. However, manually designed fusion strategies currently exhibit inherent limitations in terms of their generalization capability, failing to adapt dynamically to different lead configurations.

## 3. Materials and Method

### 3.1. Overview of Our Work

**Figure 1:**
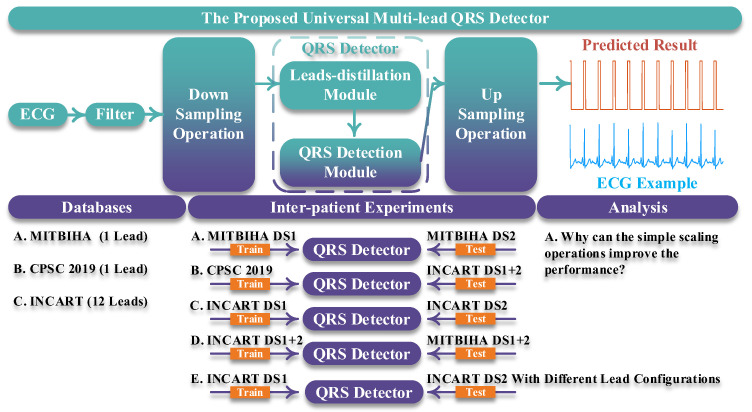
Illustration diagram of our work.

Figure 1 shows an overview of our work. In part “the proposed universal multi-lead QRS detector”, the length of the input ECG signal is designed as being 10 s. Then, the 10 s ECG signal is filtered by a band-pass filter whose lower pass-band frequency and upper pass-band frequency are 1 Hz and 40 Hz, respectively [12]. Subsequently, a simple scaling operation is conducted on the filtered ECG signal and the output of the QRS detector, corresponding to the downsampling operation and the upsampling operation in Figure 1. The scaling factor is 0.2. For example, suppose that the length of the ECG signal is 3600 sampling points; after downsampling the ECG signal with the scaling factor of 0.2, the length becomes 720 sampling points. Similarly, the length of the output of the QRS detector is upsampled to 5 times (1/0.2). Finally, in the predicted result, the QRS complexes are predicted with values of 1, and other areas are predicted with values of 0; thereby the QRS complexes are detected. The downsampling operation and the upsampling operation are quite important for our QRS detector for the following two reasons:

(1) These two operations can largely shorten the length of the input signal, thereby decreasing the computational load.
(2) These two operations can remove redundant information from the input signal, thereby improving the performance of the QRS detector.

In the “Databases” part, we employed three open-access databases to evaluate the proposed QRS detector. These are the MITBIH Arrhythmia (MITBIHA) database [13], the China Physiological Signal Challenge 2019 (CPSC2019) database [14], and the St Petersburg INCART 12-lead Arrhythmia (INCART) database [15]. The details of these databases are described in the “Database” part of this section.

In the “Inter-patient Experiments” part, we designed five experiments to evaluate our approach and compared our approach with other state-of-the-art ones. These experiments gave a comprehensive evaluation of the proposed QRS detector, including the in following areas:

(1) The performance of the QRS detector when it was trained using single-lead signals and tested using single-lead signals.
(2) The performance of the QRS detector when it was trained using single-lead signals and tested using 12-lead signals.
(3) The performance of the QRS detector when it was trained using 12-lead signals and tested using 12-lead signals.
(4) The performance of the QRS detector when it was trained using 12-lead signals and tested using single-lead signals.
(5) The performance of the QRS detector when it was trained using 12-lead signals and tested using signals using different lead configurations.

In the “Analysis” part, we additionally designed an experiment to explore the selection of the scaling factor and the reasons for the effectiveness of the scaling operation. In the procedure of exploring suitable scaling factors, we trained and tested our network with different kernel sizes of the input layer and different scaling factors. In this procedure, we only used MITBIHA DS1a and DS1b. The experimental results show that the network performs best when the scaling factor is 0.2. Therefore, we selected the scaling factor of 0.2 as the appropriate one and used it in all experiments. The relevant experimental results are displayed in Section 4. To explore the underlying reasons as to why the scaling operation can improve network performance, we also conducted a further analysis of the signals from a frequency-domain perspective. The corresponding results are also displayed in Section 4.

### 3.2. Leads-Distillation Module

**Figure 2:**
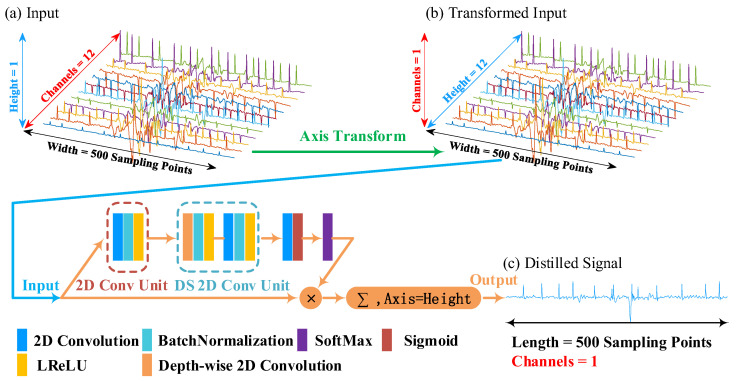
A schematic diagram of the leads-distillation module. LReLU: leaky rectified linear.

We give an example to illustrate the principle of the leads-distillation module (LDM), as shown in Figure 2. In this example, we employ a 12-lead signal with a 250 Hz sampling frequency as the input ECG signal. Because the duration of the input signal is 10 s, the length of the input is 2500 sampling points. The input ECG signal is processed through the simple scaling operation, and the length of the input ECG signal becomes 500 sampling points. Because the channel number of a convolution unit is related to that of its input feature map, once the structure of the network has been designed, the channel number of the input cannot be modified [16,17]. To realize the function of processing signals with different numbers of leads, we convert the multi-lead signal to a picture that has a large width, a small height, and one channel, as shown in Figure 2b. We call this procedure the “Axis Transform”, as shown by the green arrow of this figure.

We designed a structure to distill the multi-lead signal into a single-lead one. Firstly, we defined a 2D convolutional unit (2D Conv Unit) to extract features from the transformed input, which consists of three operations: (1) a 2D convolution with n 1 × m kernels and a “same” padding model, (2) a batch normalization (BN) to accelerate gradient propagation [18], and (3) a leaky rectified linear unit (LReLU) to conduct non-linear functions [19]. This 2D Conv Unit is denoted as follows:

(1) $$F\left(X_{H\times W\times C}\right)=\delta \left(B\left(\omega \times X_{H\times W\times C}+b\right)\right),$$

where *ω* and *b* are the weights and biases of the 2D convolution. *B*(·) denotes the batch normalization operation. *δ*(·) denotes the leaky rectified linear unit, which is defined as follows:

(2) $$\delta (X_{H\times W\times C})=\max(0,X_{H\times W\times C})+\alpha \times \min(0,X_{H\times W\times C}),$$

where *α* is the negative slope and is set to 0.3 in this work.

$X_{H\times W\times C}$ is a three-dimensional input feature map, and the superscripts H, W, and C denote the height, the width, and the number of channels, respectively. It is defined as follows:

(3) $$X_{H\times W\times C}=\left[\begin{array}{cccc} x_{1,1}^{c} & x_{1,2}^{c} & \ldots & x_{1,W}^{c}\\ x_{2,1}^{c} & x_{2,2}^{c} & \ldots & x_{2,W}^{c}\\ \vdots & \vdots & \ddots & \vdots \\ x_{H,1}^{c} & x_{H,2}^{c} & \ldots & x_{H,W}^{c} \end{array}\right],x_{i,j}^{c}\in \mathbb{R},c=1\ldots C,$$

where H, W, and C denote the height, the weight, and channels of the activation map, respectively. $x_{i,j}^{c}$ denotes a value in an activation map of channel c.

Suppose that the input is $X_{H\times W\times 1}$. The 2D Conv Unit with N convolutions outputs a feature map $Z_{H\times W\times N}$, which has N channels and can be denoted as follows:

(4) $$Z_{H\times W\times N}=\left[z^{1},z^{2},\ldots ,z^{N}\right]=F(X_{H\times W\times 1}),$$

where $z^{i}$ is the feature map of channel i.

Secondly, we employed a depth-wise separable 2D convolution unit (DS 2D Conv Unit) [20] to further extract features with a small number of parameters and low computational cost. The DS 2D Conv Unit contains two essential components, the depth-wise 2D convolution, and the point-wise 2D convolution. The depth-wise 2D convolution outputs a feature map $Y_{H\times W\times N}$, which has N channels and can be denoted as follows:

(5) $$Y_{H\times W\times N}=\left[y^{1},y^{2},\ldots ,y^{N}\right],y^{i}=\omega \times z^{i}+b,i=1,2,\ldots ,N.$$

The remaining layers of the DS 2D Conv Unit are conducted on $Y_{H\times W\times N}$ and output the feature map ${Y^{\prime }}_{H\times W\times N}$, which is defined as follows:

(6) $${Y^{\prime }}_{H\times W\times N}=F\left(\delta \left(B\left(Y_{H\times W\times N}\right)\right)\right).$$

Thirdly, we utilized a 2D convolution and an activation function “Sigmoid” to squeeze channels from N to 1 and obtained a weight matrix $A_{H\times W\times 1}$ which has one channel and can be shown as follows:

(7) $$\begin{array}{l} A_{H\times W\times 1}=Sigmoid\left(\omega \times {Y^{\prime }}_{H\times W\times N}+b\right)\\ =\left[\begin{array}{cccc} a_{1,1} & a_{1,2} & \ldots & a_{1,W}\\ a_{2,1} & a_{2,2} & \ldots & a_{2,W}\\ \vdots & \vdots & \ddots & \vdots \\ a_{H,1} & a_{H,2} & \ldots & a_{H,W} \end{array}\right],a_{i,j}\in (0,1). \end{array}$$

In this matrix, the width and height are the same as the input $X_{H\times W\times 1}$. Furthermore, each value of this matrix denotes the weight of each sampling points of $X_{H\times W\times 1}$.

To obtain the important sampling points among all leads, we conducted the operation “SoftMax” on each column of the matrix $A_{H\times W\times 1}$. The procedure can be expressed as follows:

(8) $${a^{\prime }}_{i,j}=\frac{e^{a_{i,j}}}{\sum_{k=1}^{H}e^{a_{k,j}}},j=1,2,\ldots ,W,i=1,2,\ldots ,H$$

(9) $${A^{\prime }}_{H\times W\times 1}=\left[\begin{array}{cccc} {a^{\prime }}_{1,1} & {a^{\prime }}_{1,2} & \ldots & {a^{\prime }}_{1,W}\\ {a^{\prime }}_{2,1} & {a^{\prime }}_{2,2} & \ldots & {a^{\prime }}_{2,W}\\ \vdots & \vdots & \ddots & \vdots \\ {a^{\prime }}_{H,1} & {a^{\prime }}_{H,2} & \ldots & {a^{\prime }}_{H,W} \end{array}\right],{a^{\prime }}_{i,j}\in \left(0,1\right).$$

We multiplied the matrix ${A^{\prime }}_{H\times W\times 1}$ and the input $X_{H\times W\times 1}$ to weaken the value of unimportant sampling points of $X_{H\times W\times 1}$, which can be denoted as follows:

(10) $${X^{\prime }}_{H\times W\times 1}=X_{H\times W\times 1}\cdot {A^{\prime }}_{H\times W\times 1}.$$

Finally, we obtained the distilled signal S by summing the values of each column in ${X^{\prime }}_{H\times W\times 1}$, which can be denoted as follows:

(11) $$s_{i}=\sum_{k=1}^{H}{x^{\prime }}_{k,i},i=1,2,\ldots ,W,$$

(12) $$S=\left[s_{1},s_{2},\ldots ,s_{W}\right],s_{i}\in \mathbb{R},$$

where $s_{w}$ is the sampling point of the distilled signal.

**Figure 3:**
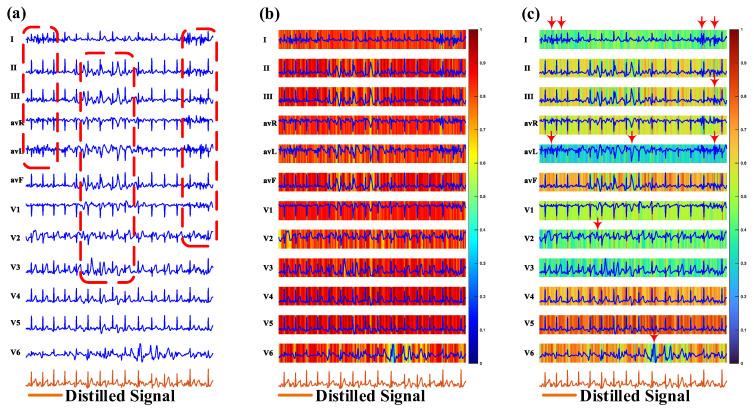
A visualization of the leads-distillation module (LDM). (**a**) 12-lead signal; (**b**) feature map before the SoftMax operation; (**c**) feature map after the SoftMax operation.

To gain a deeper understanding of the leads-distillation module (LDM), we visualized the 12-lead ECG signals, the feature maps before and after the SoftMax operation, and the distilled signal, as shown in Figure 3. In Figure 3a, there are some noises near the QRS complexes in most leads, and there are even some fake QRS complexes, as shown in the red regions. In stark contrast, the power of these noises is weakened in the distilled signal. Figure 3b shows the weights of each sampling point of the 12-lead signal, but the important sampling points of each lead are not obvious. Figure 3c shows the result output from the SoftMax operation, and the important sampling points of each lead are more obvious. In this figure, the weights of some noises, like a QRS complex, are attenuated, as shown in positions indicated by arrows. Furthermore, we list the hyperparameters of the LDM in Table 1.

### 3.3. QRS Detection Module

**Figure 4:**
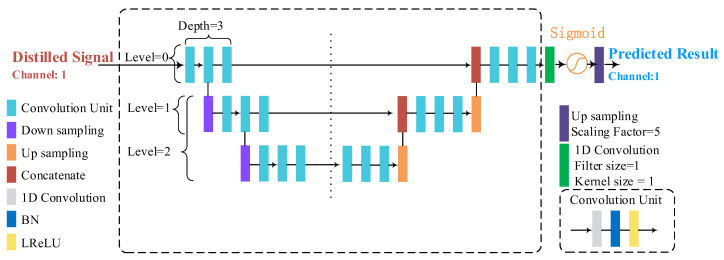
A schematic diagram of the QRS detection module. BN is the abbreviation for batch normalization. LReLU is the abbreviation for the leaky rectified linear unit.

We designed a framework to further detect QRS complexes from the distilled signals, named the QRS detection module, as shown in Figure 4. The prototype of the proposed framework comes from the U-Net, which was proposed by Ronneberger et al. in the top conference “MICCAI” for the first time and was used for biomedical image segmentation [21]. The hyperparameters of the QRS detection module are listed in Table 2, including the depth, filter size, number of levels, and kernel size of each level.

In Figure 4, the proposed framework shows a left and right symmetrical structure. The length of input and output is the same as that of the distilled signal. We regard each horizontal path as the level and regard the number of convolution units on the left side as the depth of each horizontal path. The depth of all levels and the filter size of all convolution units are uniform in the whole framework. The kernel size of all convolution units in each level is also uniform. The operation “Down sampling” can reduce the length of the inputted feature map by half. Correspondingly, the operation “Up sampling” can double the length of the inputted feature map. The operation “Concatenate” is used to concatenate inputted feature maps by channel. If no feature map came from the lower level, the operation “Concatenate” of the current level can be omitted. The final activation function is “Sigmoid”. Subsequently, an upsampling operation is conducted five times on the output of the final activation function. It is also a part of the simple scaling operation.

### 3.4. Training Environments and the Loss Function

The proposed QRS detector was developed in the platform “Pytorch” with the random seed “0”. The hardware platform was Nvidia RTX2080Ti. The QRS detector was trained with dice loss [22], the Adam optimizer [23], a learning rate of 0.0001, and 100 epochs.

We utilized dice loss as the loss function. Suppose that the predicted result is $P=\{p_{1},p_{2},\ldots ,p_{n}\}$ and the target is $T=\{t_{1},t_{2},\ldots ,t_{n}\}$. We calculate the dice coefficient as follows:

(13) $$D_{coefficient}=\frac{2\sum_{i}^{N}p_{i}\cdot t_{i}}{\sum_{i}^{N}p_{i}^{2}+\sum_{i}^{N}t_{i}^{2}+\delta },$$

where $p_{i}$ is the point of the predicted result and $t_{i}$ is the point of the true label. In this work, we set δ as 0.00001.

Then, we can calculate the dice loss as follows:

(14) $$DiceLoss=1-D_{coefficient}.$$

### 3.5. Database

In this work, we employ three open-access databases to evaluate the proposed QRS detector and study our findings, as shown in Table 3.

The MITBIH Arrhythmia (MITBIHA) database includes 48 half-hour two-channel recordings. These recordings were acquired from the modified limb leads II (ML II) and V (typically V2) with a sampling rate of 360 Hz [13]. We only used signals acquired from ML II. Following the recommendation of ANSI/AAMI EC57:1998 standards, four recordings that have the pacemaker are not used in this paper; they are recordings 102, 104, 107, and 217. The data distribution of DS1 and DS2 was proposed by De Chazal et al. and is widely used by other researchers [24]. Moreover, we further divided the dataset “DS1” into two datasets, namely DS1a and DS1b. They were used to investigate our findings and explore the scaling factor for the simple scaling operation.

The China Physiological Signal Challenge 2019 (CPSC2019) database contains 2000 ECG recordings. Each recording has a duration of 10 s and a sampling frequency of 500 Hz [14]. The ECG signals of this database have only one channel and are noisier than the MITBIHA database [25].

The St Petersburg INCART 12-lead Arrhythmia (INCART) database has 75 annotated recordings extracted from 32 Holter recordings [15]. Each recording is 30 min long and has 12 channels. The signals of this database were sampled at 257Hz. The data distribution of DS1 and DS2 was proposed by Víctor Mondelo et al. [26]. This database was also used to evaluate the performance of our method with different configurations, including six leads, four leads, three leads, two leads, and a single lead. For this purpose, the DS1 was used to train and keep the original number of leads, while the test dataset “DS2” removes irrelevant leads according to the corresponding lead configuration.

**Figure 5:**
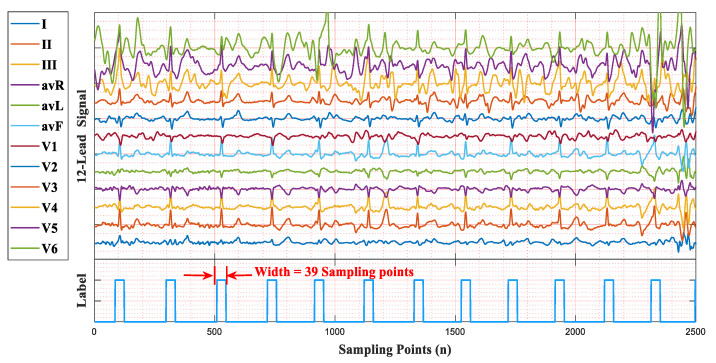
An example of the QRS label.

We used a 12-lead ECG signal with a sampling frequency of 257 Hz to illustrate the QRS label, as shown in Figure 5. The area of a QRS complex is labeled with values of 1, the width is 39 sampling points (19 sampling points before and after an R-peak), and other areas maintain values of 0. The duration of a QRS label is about 150ms, which was selected following the standards of the Association for the Advancement of Medical Instrumentation (AAMI) [27]. Similarly, for 360 Hz, the width of a QRS label is 55 sampling points, while it is 75 sampling points for 500 Hz.

## 4. Experimental Results

### 4.1. Evaluation Metrics

To compare the performance with other methods and evaluate our method, we used three evaluation metrics, namely sensitivity (Se), positive predictive value (PPv), and F1 score. These evaluation metrics are defined as follows:

(15) $$Se=\frac{TP}{TP+FN}\times 100\%,$$

(16) $$PPv=\frac{TP}{TP+FP}\times 100\%,$$

(17) $$F1\;score=\frac{2\times TP}{2\times TP+FP+FN}\times 100,$$

where TP, FP, and FN are the number of true-positive QRS complexes, the number of false-positive QRS complexes, and the number of false-negative QRS complexes, respectively.

According to the AAMI standard [27], for a true-positive QRS complex, the detected QRS complex should lie in a region of a duration of 75 ms centered on the reference QRS annotation. The false-positive QRS complex refers to the detected QRS complex out of the 75 ms region centered on the reference QRS annotation. Furthermore, it is a false-negative QRS complex if there is no detected QRS complex in a region of duration 75 ms centered on the annotation.

### 4.2. Results of Single-Lead Training and Single-Lead Testing

We compared our approach with two excellent methods, as shown in Table 4. These methods are also based on the convolutional neural network. And these methods were trained and tested with the same databases as ours. It is obvious that our method (with scaling factor 0.2) achieves the best performance.

It is worth noting that the scaling factor of 1 means that we do not scale the input ECG signal. When we scale the input ECG signal with the scaling factor 0.2, the F1 score increases from 98.60 to 99.83. This demonstrates that the scaling operation can improve the QRS detection performance.

### 4.3. Results of Single-Lead Training and 12-Lead Testing

The result of single training and 12-lead testing is shown in Table 5. The compared methods were also developed with the convolutional neural network, while the training dataset and testing dataset were the same as ours. For the result of the method proposed by Cai et al. [29], although the original paper did not test their network on the INCART database, Liu et al. [30] tested this network on this database. For results 1 to 4 in Table 5, our method achieves the best F1 score of 99.22 compared to other approaches. Results 3 and 4 again demonstrate the effectiveness of the simple scaling operation.

Due to the compatibility of the LDM, the trained network can be used directly on the single lead signal without any changes being made to the network. Results 5 to 16 show the performance of our proposed method with each lead. The best score appears with lead V1, and the worst one appears with lead I. Although the performance of our method with all leads does not surpass the performance on lead V1, the close result still can demonstrate that our method can fuse useful information from multiple leads.

It is worth noting that there is no way to know which lead can provide the best performance in practice. Therefore, if a QRS detector is designed only for a single lead, it may not achieve the best performance. This is the limitation of the QRS detector designed only for a single lead.

### 4.4. Results of 12-Lead Training and 12-Lead Testing

In this part, we cited an excellent method to compare with the proposed method, which is the one proposed by Victor et al. [26]. Although this cited paper does not employ deep learning technology, it is the only paper that tests using the same data distribution as ours. For this dataset, our method (with the scaling factor 0.2 for all leads) obtained an F1 score of 99.77 and the best sensitivity of 99.88%. Comparing result No. 2 with result No. 3, the F1 score increases by about 0.81, which also demonstrates the effectiveness of the simple scaling operation.

For single-lead results 4 to 15, the best score appears with lead V6 and the worst one appears with lead V2, as shown in Table 6. The result of our method for all leads reaches an F1 score of 99.77. This demonstrates the effectiveness of our method in fusing useful information from multiple leads.

### 4.5. Results of 12-Lead Training and Single-Lead Testing

The result of 12-lead training and single-lead testing is shown in Table 7. In this table, we cited another method to compare with our method. It was proposed by Habibi et al. [31]. The cited method was designed with the convolutional neural network and evaluated using the same databases as ours. Among these results, the sensitivity, the positive predictive value, and the F1 score of our method (with scaling factor 0.2) surpass the other results, achieving the best performance of QRS detection.

Because the cited method was trained with channel I of the “INCART (DS1+DS2)” database, we also carried out the same experiment, and the result is shown as result No. 4 of Table 7. This result still surpasses the cited paper. Moreover, the F1 score of result No. 3 is higher than that of result No. 4. This is because our network can fuse useful information from other leads.

### 4.6. Results of 12-Lead Training and Multi-Lead Testing

Because there are few QRS detectors designed with the convolutional neural network for the multi-lead ECG signals, we only reproduced a suitable network to compare with the proposed method. It was proposed by Viktor et al. [32]. There are some reasons for this. The first reason is that this reproduced network was designed with a convolutional neural network. The second reason is that although the authors designed this network for single-lead signals, they provided a method to handle multi-lead signals, which involves using the network to identify QRS complexes from each lead independently and then calculating the average of the predicted results. The last reason is that although this network was designed to delineate P waves, T waves, and QRS complexes, the delineating style is the same as our QRS detecting style, which is the point-wise classification. Moreover, we only retained one output channel of this produced network for QRS detection. The experimental results are shown in Table 8. In this table, our method achieves the best performance for the six-lead, four-lead, two-lead, and single-lead configurations. For the three-lead configuration, the f1 score had a difference of just 0.29.

### 4.7. Results of the Scaling Operation Experiment

In the process of searching for the appropriate scaling factor, we selected five scaling factors, as shown in Table 9. We selected them with some limited conditions, as follows:

(1) Let us take 1 as the dividend and the scaling factor as the divisor. The value of 1 must be divided evenly.
(2) The minimum scaling factor is 0.1.

**Figure 6:**
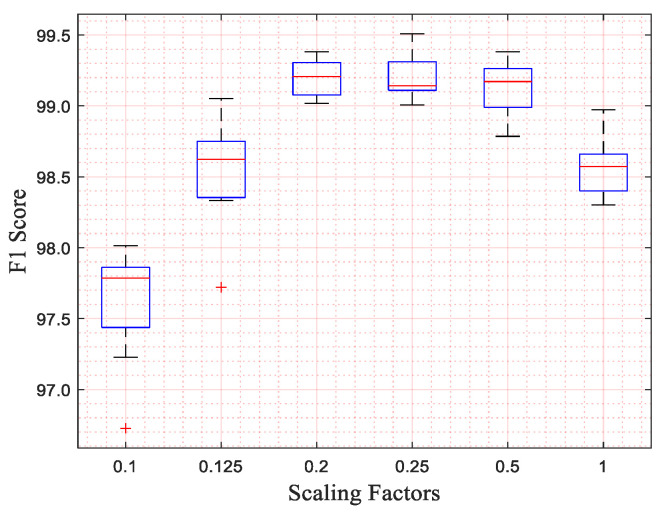
The performance of our network with different scaling factors and kernel sizes.

The F1 scores of different scaling factors are plotted as box plots, as shown in Figure 6. The results of different kernel sizes for the same scaling factor are grouped into the same box, where the upper and lower blue edges represent the upper and lower quartiles, respectively; the red line indicates the median; and the upper and lower black edges represent the maximum and minimum values, respectively. In this figure, the performance of the network for the scaling factors 0.1, 0.125, and 1 has obvious differences compared to the scaling factors 0.2, 0.25, and 0.5. Because the difference between the top line and bottom line for the scaling factors of 0.25 and 0.5 is bigger than that of the scaling factor of 0.2, the network with the scaling factors of 0.25 and 0.5 has poor stability. Therefore, we regarded the scaling factor 0.2 as the suitable one and used it.

**Figure 7:**
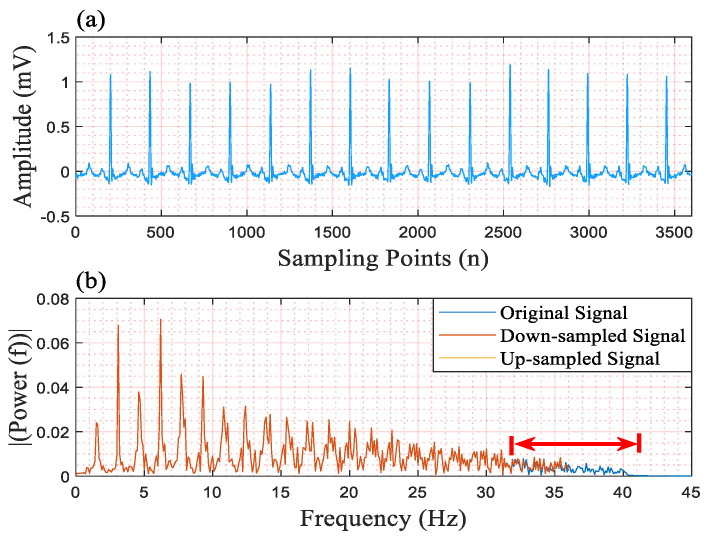
The change in the frequency component between the original signal and the scaled signal. (**a**) the filtered signal; (**b**) the result of FFT.

Regarding the effectiveness of the scaling operation, we think that certain frequency components of the input signal are not conducive to our network’s detection of QRS complexes, and the simple scaling operation can easily remove these components. To this end, we downsampled the input signal with scaling factors of 1 and 0.2, and then we upsampled the downsampled signal to the original length using the nearest values. We give an FFT example to illustrate the changes in the frequency component, as shown in Figure 7. In this example, the signal is downsampled with the scaling factor 0.2, and then the downsampled signal is upsampled five times (1/0.2) to one with the original length. In this figure, (a) is the filtered signal, and (b) is the result of FFT. In Figure 7b, we only show the power between 0.1 Hz and 45 Hz. The area below the red arrow is the main change in the frequency component. The power curves of the downsampled signal and the upsampled signal coincide. This proves that the downsampling operation and the upsampling operation can remove the higher frequency component, and that the approach of upsampling signals using the nearest values adds no other extra frequency component.

Subsequently, we take processed signals into the network, and the result are shown in Table 10. Among these results, the F1 score decreases when the scaling factor is set to 1, 0.5, and 0.25, respectively. When the scaling factor is set to 0.2, the F1 score reaches the best value. The F1 score then continues to decrease. This demonstrates that the frequency component between 36 Hz and 40 Hz is not conducive to our network when detecting QRS complexes. With the removal of the lower frequency component, the detection performance decreases. This may be caused by the excessive removal of the frequency component.

## 5. Discussion

In this paper, we proposed a novel multi-lead QRS detector based on convolutional networks involving no manual strategies. In contrast to methodologies reliant on manual strategies, our approach exhibits heightened robustness and generalizability when faced with intricate and noise-laden signals. It has been thoroughly substantiated by the experiments presented earlier in the text. We employed a methodology involving single/multi-lead training and single/multi-lead testing to evaluate the generalizability of our approach in the face of unknown quantities of lead data. Compared with the state-of-the-art methods, the method proposed in this paper shows considerable improvements in sensitivity and also achieves better or comparable results in terms of PPV and F1 score.

Within our research, we have observed that the comparative methods showcased subpar performance when the number of training leads differed from the number of test leads. For example, in the experiment of single-lead training and 12-lead testing, the methods proposed by Cai et al. [29] and Liu et al. [30] achieved F1 scores of 95.68 and 92.90, respectively, falling short compared to the results achieved by our method. In our opinion, there are two potential factors that could account for this phenomenon.

(1) Methods with manual strategies exhibit significant variations in performance when applied to diverse datasets involving different leads. A recent study by Chauhan serves as an example, where the author meticulously devised a multi-lead fusion strategy that effectively excluded anomalies present in only a few leads [33]. This approach showcased impressive results within the twelve-lead database. However, when applied to datasets with fewer leads, the reduction in leads resulted in an increased weightage of QRS wave errors. Consequently, when applied to a single lead, devoid of information from other leads, the fusion strategy loses its capability to eliminate errors.
(2) Our method, which detects the QRS complex with substitute signals, renders our method less susceptible to variations in input data compared to other methods. Even in practical applications where the number of leads used may differ from the training phase, our LDM module is capable of generating signals that are most favorable for the QRS detector. These generated signals exhibit similar features.

Furthermore, we have observed an intriguing phenomenon: simple scaling operations can enhance the performance of our QRS detector. We conducted experiments and provided an explanation for this phenomenon in Section 4.7. The results show that simple scaling operations can eliminate specific frequency components in the signal that are detrimental to network processing, thereby improving the network’s performance.

There are some limitations to be resolved in our future work. Firstly, in the third experiment, the performance of the proposed network in this paper did not surpass that of the compared network in terms of PPV and F1 score. Therefore, our method still has room for improvement. Secondly, the scaling factor of 0.2 was selected using the “MITBIHA” database. Although this scaling factor is effective for the other two databases, these databases may have a more suitable scaling factor. The relationship between the sampling frequency and the suitable scaling factor is still unclear. We will explore this question in our next work. Thirdly, the scaling factor was selected manually. This may cause generalization problems in other databases. In our next work, we plan to propose a novel module to select the scaling factor automatically.

## 6. Conclusions

In this paper, we propose a novel QRS detector that does not involve any manual strategies for fusing multiple leads and has good compatibility with multi-lead signals. Our QRS detector consists of two modules, namely the LDM and the QRS detection module. The LDM is designed to distill multiple signals into one signal. The QRS detection module detects QRS complexes from the distilled signal. Moreover, we found an interesting phenomenon in which downsampling the input signal appropriately can improve the capability of our network to detect QRS complexes. We designed five experiments to evaluate the performance of our network. The result shows that the proposed approach has good performance. Furthermore, we also explain why the scaling operation can improve the QRS detector’s performance. The reason is that the scaling operation can remove some frequency components that are not conducive to our network’s detection of QRS complexes.

In future work, we will design a novel structure to improve the QRS detection performance and automatically select scaling factors. Moreover, we will investigate the relationship between sampling frequency and the most suitable scaling factor in our next work.

The significance of this work is that this approach circumvents the need for the repeated deployment of the QRS detection function to different lead configurations in the ECG diagnostic system. Moreover, the scaling operation may become a simple tool to decrease the computational load of the network.

## Figures and Tables

**Table 1:** The hyperparameters of the LDM.

Group	Name	Filter Size	Kernel Size
2D Conv Unit	2D Convolution	13	1 × 11
	Batch normalization	-	-
	Leaky rectified linear unit	-	-
DS 2D Conv Unit	Depth-wise 2D convolution	1	1 × 11
	Batch normalization	-	-
	Leaky rectified linear unit	-	-
	Point-wise 2D convolution	13	1 × 1
	Batch normalization	-	-
	Leaky rectified linear unit	-	-
-	2D convolution	1	1 × 11
-	Sigmoid	-	-
-	SoftMax	-	-

**Table 2:** The hyperparameters of the QRS detection module.

Hyperparameters	Value	Hyperparameters	Value
Filter size	3	Kernel size (level 0)	11
Depth	3	Kernel size (level 1)	17
Level	2	Kernel size (level 2)	13

**Table 3:** Databases and the data distribution.

MITBIH Arrhythmia (MITBIHA) 1-Lead				
Number of Recordings			Total Heartbeats	Sampling Frequency
DS1	DS1a	DS1b	100,718	360 Hz
	101,106,108,109,112,114,115,116,118,119,122	124,201,203,205,207,208,209,215,220,223,230		
DS2	100,103,105,111,113,117,121,123,200,202,210,212,213,214,219,221,222,228,231,232,233,234			
**China Physiological Signal Challenge 2019 (CPSC2019) 1-Lead**				
-			29,467	500 Hz
**St Petersburg INCART 12-lead Arrhythmia (INCART) 12-Lead**				
DS1	I01-I39 (39 Recordings)		175,911	257 Hz
DS2	I40-I75 (36 Recordings)			

**Table 4:** The Result on MITBIH DS2.

Method	Se (%)	PPv (%)	F1 Score
He 2021 [25]	99.56	99.72	99.63
Belkadi 2021 [28]	99.76	99.24	99.50
Proposed Method (* SF 1)	99.89	97.35	98.60
Proposed Method (SF 0.2)	**99.92**	**99.74**	**99.83**

* SF: an abbreviation of the scaling factor.

**Table 5:** The result on the INCART database (DS1 + DS2).

No.	Method	Se (%)	PPv (%)	F1 Score
1	Liu 2020 [30]	94.99	96.37	95.68
2	Cai 2020 [29]	97.07	89.06	92.90
3	Proposed method (SF 1, all leads)	98.97	99.24	99.10
4	Proposed method (SF 0.2, all leads)	**99.14**	**99.29**	**99.22**
5	Proposed method (SF 0.2, lead I)	96.18	91.76	**93.91**
6	Proposed method (SF 0.2, lead II)	99.32	97.92	98.62
7	Proposed method (SF 0.2, lead III)	99.12	97.84	98.48
8	Proposed method (SF 0.2, lead avR)	98.92	96.81	97.85
9	Proposed method (SF 0.2, lead avL)	97.68	95.42	96.53
10	Proposed method (SF 0.2, lead avF)	99.31	98.69	99.00
11	Proposed method (SF 0.2, lead V1)	99.35	99.28	**99.31**
12	Proposed method (SF 0.2, lead V2)	99.38	99.00	99.20
13	Proposed method (SF 0.2, lead V3)	98.04	99.13	98.58
14	Proposed method (SF 0.2, lead V4)	97.58	99.34	98.45
15	Proposed method (SF 0.2, lead V5)	99.37	98.98	99.18
16	Proposed method (SF 0.2, lead V6)	97.87	98.96	98.41

**Table 6:** The results on the INCART DS2 database.

No.	Method	Se (%)	PPv (%)	F1 Score
1	Víctor 2017 [26]	99.86%	**99.98%**	**99.95**
2	Proposed method (SF 1, all leads)	99.55	98.38	98.96
3	Proposed method (SF 0.2, all leads)	**99.88**	99.65	99.77
4	Proposed method (SF 0.2, lead I)	95.64	93.99	94.81
5	Proposed method (SF 0.2, lead II)	96.37	93.21	96.37
6	Proposed method (SF0.2, lead III)	99.59	91.65	95.45
7	Proposed method (SF 0.2, lead avR)	99.24	94.67	96.90
8	Proposed method (SF 0.2, lead avL)	93.58	93.96	93.77
9	Proposed method (SF 0.2, lead avF)	99.68	93.15	96.31
10	Proposed method (SF 0.2, lead V1)	99.76	94.82	97.23
11	Proposed method (SF 0.2, lead V2)	99.68	93.41	**93.13**
12	Proposed method (SF 0.2, lead V3)	99.73	94.56	97.07
13	Proposed method (SF 0.2, lead V4)	95.66	96.74	96.19
14	Proposed method (SF 0.2, lead V5)	99.72	96.86	98.26
15	Proposed method (SF 0.2, lead V6)	99.77	97.62	**98.68**

**Table 7:** The results on the MITBIHA database (DS1 + DS2).

No.	Method	Se (%)	PPv (%)	F1 Score
1	Habib 2019 [31]	97.61	91.93	94.68
2	Proposed method (SF 1)	99.89	93.21	96.43
3	Proposed method (SF 0.2)	**99.92**	**98.28**	**99.09**
4	Proposed method (SF 0.2) *	99.88	97.31	98.58

* A special result. This result comes from our network trained with channel I of the 12-lead signals in the “INCART (DS1 + DS2)” database.

**Table 8:** F1 score for multiple lead configurations.

		Number of Leads					
No	Method	6	4	3	2	1	Number of Parameters
1	Viktor 2020 [32]	98.67	98.60	**99.23**	96.83	96.29	167,329
2	Proposed method (SF 0.2)	**99.68**	**99.54**	98.94	**98.51**	**96.38**	**5216**

**Table 9:** The parameters of the exploratory experiment.

Scaling Factors	1	0.5	0.25	0.2	0.125	0.1
Kernel Size	1,3,5,7,9,11,…,17,19					

**Table 10:** The results of different scaling factors.

Scaling Factors	1	0.5	0.25	0.2	0.125	0.1
F1 score	98.97	98.75	98.74	99.21	98.93	95.51

## Data Availability

The CPSC2019 dataset is available at http://2019.icbeb.org/Challenge.html, accessed on 22 May 2025. The MITBIHA dataset is available at https://www.physionet.org/content/mitdb/1.0.0/, accessed on 22 May 2025. The INCART dataset is available at https://physionet.org/content/incartdb/1.0.0/, accessed on 22 May 2025.
